# EHMTI-0367. Comparison of sleep quality and migraine headaches in people with proper and improper and poor sleep

**DOI:** 10.1186/1129-2377-15-S1-J15

**Published:** 2014-09-18

**Authors:** N Torabzadeh, S Asadnia, F Sepehrian Azar, REZA Banihashemi, NAVA Mohammadi

**Affiliations:** 1 Psychology Department, Urmia University of Medical Sciences, Urmia, Iran; 2 Psychology Department, Urmia University, Urmia, Iran; 3 Psychology Department, Ardabil University, Urmia, Iran; 4 Psychology Department, Urmia, Iran

## Introduction

Sleep disorder is prevalent in patients with migraine headaches. . This research aims to compare sleep quality and migraine headaches in patients with proper and improper sleep.

## Aims

The Aim of this Study was to Comparison of sleep quality and migraine headaches in people with proper and improper and poor sleep.

## Methods

This study is both descriptive and comparative. The population included all male and female undergraduate students at the University Urmia of the 2011-2012 school year who were selected by simple random sampling .Thus, in the first step, 280 people completed Pittsburgh Sleep Quality Test and Najjarian Migraine headache symptoms questionnaire. In the next step, 115 students who scored lower than the cut point on the tests of sleep quality and 115 of the students who scored above the cut point were selected and were stratifies as people with proper and improper sleep. The test data were analyzed using multivariate variance statistical analysis.

## Results

The results showed no significant difference between the two groups in terms of the variables being studied .The group with improper sleep obtained a higher average in the variable of migraine headache, sleep quality and its sub-scales i.e. subjective sleep quality, delay and difficulty falling asleep, sleep duration, good sleep, sleep disorders, use of narcotics and sleep medications and daily dysfunctions.

## Conclusions

People who have poor sleep quality, sleep disorders, migraine headaches and difficulty falling asleep experience more problems in their life.

No conflict of interest.

